# A shorter linker in the bispecific antibody RmAb158-scFv8D3 improves TfR-mediated blood-brain barrier transcytosis in vitro

**DOI:** 10.1038/s41598-024-83627-6

**Published:** 2024-12-23

**Authors:** Inga Petersen, Jamie I. Morrison, Alex Petrovic, Neira Babic, Nicole G. Metzendorf, Ana Godec, Andrés de la Rosa, Fadi Rofo, Sina Bondza, Jos Buijs, Farahnaz Ranjbarian, Anders Hofer, Dag Sehlin, Greta Hultqvist

**Affiliations:** 1 Department of Pharmacy, Uppsala University, Uppsala, Sweden; 2 Ridgeview Instruments AB, Uppsala, Sweden; 3 Department of Immunology, Genetics and Pathology, Uppsala University, Uppsala, Sweden; 4 Department of Medical Biochemistry and Biophysics, Umeå University, Umeå, Sweden; 5 Department of Public Health and Caring Sciences, Uppsala University, Uppsala, Sweden

**Keywords:** Blood-brain-barrier (BBB) shuttle, Transferrin receptor (TfR), RmAb158-scFv8D3, Receptor crosslinking, Bispecific antibodies, Monovalent and bivalent binding, Proteins, Protein delivery, Antibody therapy, Protein design

## Abstract

Transferrin Receptor (TfR)-mediated transcytosis across the blood-brain barrier (BBB) enables the uptake of bispecific therapeutic antibodies into the brain. At therapeutically relevant concentrations, bivalent binding to TfR appears to reduce the transcytosis efficiency by receptor crosslinking. In this study, we aimed to improve BBB transcytosis of symmetric antibodies through minimizing their ability to cause TfR crosslinking. We created variants of the previously published RmAb158-scFv8D3, where the linker length between RmAb158 and the mTfR-targeting scFv8D3 was adjusted. We investigated the effect of the linker length on the antibodies’ binding kinetics to mTfR using ELISA and LigandTracer assays, and their ability to transcytose across BBB endothelial cells (In-Cell BBB-Trans assay). We show that even a direct fusion without a linker does not alter the antibodies’ apparent affinities to mTfR indicating their valency is unlikely affected by the linker length. However, the shortest linker variants demonstrated BBB transcytosis levels comparable to that of the monovalent control at a high antibody concentration and showed an almost two-fold higher level of BBB transcytosis compared to the longer-linker variants at the high concentration. Our new RmAb158-scFv8D3 short-linker variants are examples of symmetric, therapeutic antibodies with improved TfR-binding characteristics to facilitate more efficient brain uptake. We hypothesize that bivalent binding to TfR as such does not negatively affect BBB transcytosis in vitro, but a very short distance between TfR-targeting domains lowers the probability of receptor crosslinking. This study provides valuable insights into antibody-TfR interaction kinetics, contributing to future development of TfR-targeting antibody-based treatments for brain diseases.

**Supplementary Information:**

The online version contains supplementary material available at 10.1038/s41598-024-83627-6.

## Introduction

The large size and the endless variability of protein drugs enable them to bind with high specificity to their targets. Monoclonal antibodies offer particularly high specificity and affinity to their targets and have been established as therapeutic drugs in the fields of cancer and transplantations as well as autoimmune, inflammatory, cardiovascular and infectious diseases^1^. However, in contrast to small molecule drugs, the large size of protein drugs limits their application to easily accessible tissues.

The uptake of substances into the brain is particularly tightly regulated by the blood-brain barrier (BBB), formed by endothelial tight junctions, pericytes and astrocytic end-feet. Large molecules such as proteins can therefore not enter the brain passively. The proportion of antibodies that reach the interstitial fluid is estimated to be less than 0.05–0.1% of the intravenously injected dose^2,3^. A well-established approach to enable active protein drug delivery to the brain is to utilize existing endogenous receptor-mediated transport pathways, such as via the transferrin receptor (TfR)^4–7^.

TfR is a homodimeric receptor that is highly expressed on the apical surface of endothelial cells in the BBB and enables the transport of iron into the brain. However, the trafficking of iron, transferrin (Tf), and TfR-targeting antibodies through the BBB endothelial cells is not well understood. Besides being expressed on endothelial cells, TfR is also expressed on red blood cells, where the iron and Tf trafficking is much better characterized. On red blood cells, iron-loaded Tf (holoTf) binds to TfR, the complex is endocytosed, and iron dissociates from Tf in the low pH of the endosome. From the endosome, iron is actively transported into the cytosol while TfR together with Tf is recycled back to the cell membrane of the red blood cell^8^. In the case of BBB endothelial cells, it is debated whether iron dissociates from the TfR-Tf complex within the endosome or whether it is released as holoTf complex to the brain interstitium^9–12^. It has also been suggested that instead of being released by exocytosis, holoTf may be released within exosomes from the BBB endothelial cell to the brain^11^. The endogenous characteristics of TfR- and Tf-trafficking in brain endothelial cells likely determine the transcytosis route of TfR-targeting antibodies and thus poses specific requirements for the kinetics of the antibody-TfR interaction.

Antibodies binding with high affinity to TfR have been shown to cause the TfR/ligand complex to be sorted to lysosomal degradation^13^. Additionally, bivalent binding to TfR has been suggested to cause crosslinking of TfR, leading to the formation of larger TfR networks on the cell surface. Crosslinked TfR can result in slower endocytosis of the TfR/ligand complex, its retention inside the endothelial cells, lysosomal degradation and downregulation of TfR^14–17^. However, the transcytosis-limiting effects are concentration dependent. At a low antibody concentration, TfR-crosslinking seems negligible and bivalent antibodies with high affinity to TfR are more efficient than monovalent TfR binders in passing the BBB^16,18^. At higher concentrations, monovalent and low affinity TfR-binders have been shown to result in higher brain uptake compared to bivalent TfR-binders, as they do not crosslink TfRs^5,18–20^.

**Fig. 1:**
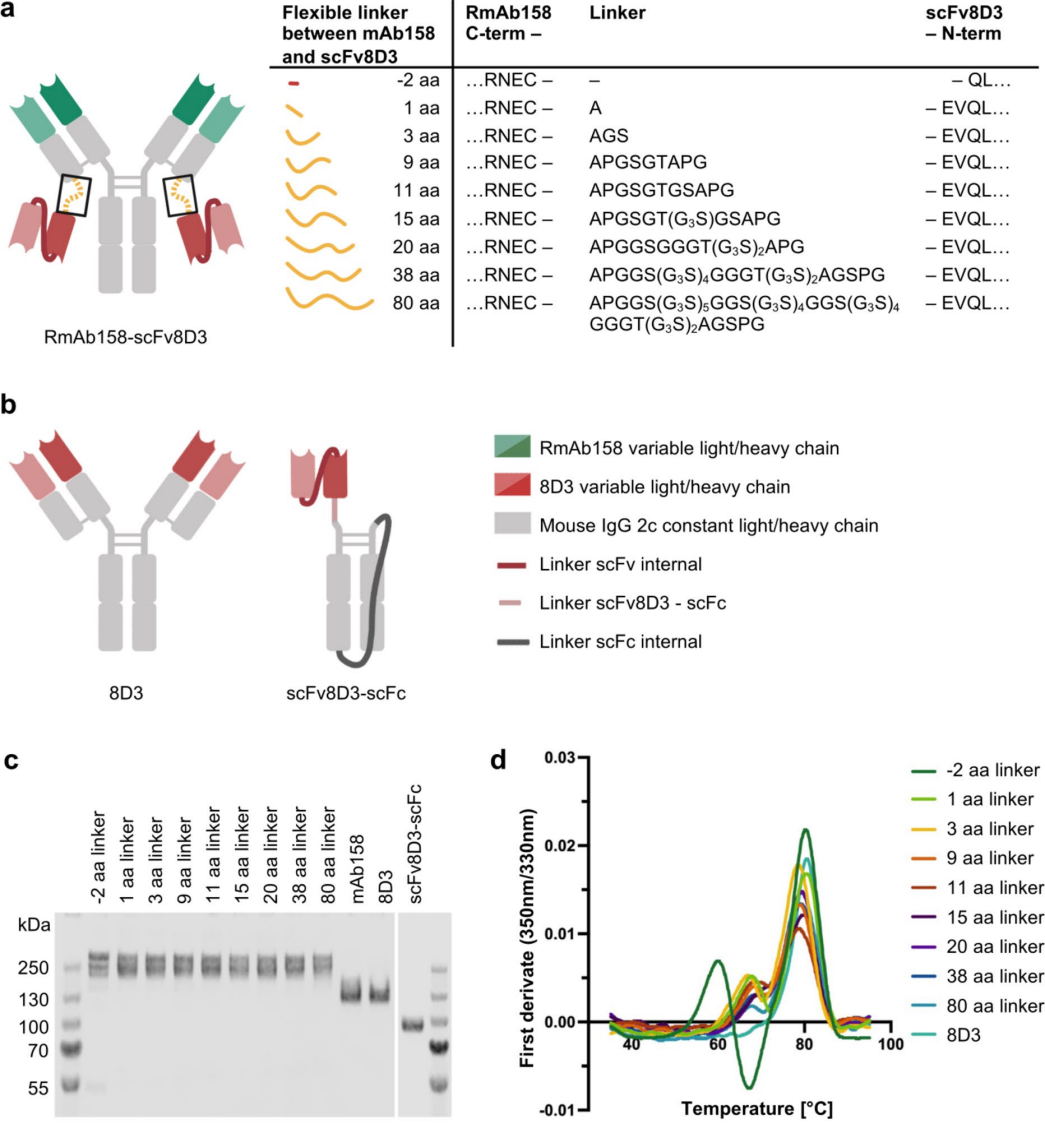
Design and structural integrity analysis of antibodies used in this study. (**a**) The original RmAb158-scFv8D3 design with an 11 aa linker between RmAb158 and scFv8D3, and the eight variants of RmAb158-scFv8D3 with only the length of the linker altered (yellow). (**b**) Antibody controls 8D3^24^ for bivalent TfR-binding and scFv8D3-scFc^16^ for monovalent TfR-binding. (**c**) SDS-PAGE with Coomassie staining of the purified RmAb158-scFv8D3 variants and control antibodies under non-reducing conditions. 1 µg protein/lane. Complete gel and SDS-PAGE under reducing conditions can be found in Supplementary Fig. S2a,b. (**d**) Thermal stability of purified RmAb158-scFv8D3 variants and 8D3 measured by the change of the antibodies’ intrinsic fluorescence at 330 nm and 350 nm over a temperature ramp. Inflection temperatures, represented as peaks in the first derivate of the fluorescence intensity ratio 350 nm/330 nm, indicate major unfolding events. Raw data can be seen in Supplementary Fig. S2c and Table S1.

Bivalent binding of an antibody to its target, in contrast to monovalent binding, increases its overall binding strength due to the avidity effect, which is the combined affinity of both antibody arms^21^. The higher valency primarily affects the dissociation rate, because once the antibody has bound with both arms, it strongly decreases the chances of the antibody to fully dissociate from its target. Most monovalent TfR-targeting antibodies described in publications are asymmetric in design^6,19,20^. The antibody RmAb158-scFv8D3 was previously developed in our lab as a symmetric, bispecific antibody, which can efficiently cross the BBB^5^. It is based on the full-sized amyloid-β (Aβ) protofibril-targeting antibody mAb158^22,23^ with single-chain variable fragments (scFv) of the murine TfR1 (mTfR)-targeting antibody 8D3^24^ at the C-terminal end of both antibody light chains (Fig. 1a). The scFv8D3 are attached recombinantly through flexible linkers of 11 amino acids (aa), which was considered short enough to sterically hinder the two scFv8D3 from crosslinking two mTfR subunits on the cell surface. RmAb158-scFv8D3 showed 80-fold higher brain uptake compared to RmAb158 alone at lower tracer doses and a 10-fold lower binding strength to mTfR compared to the full 8D3 in a competition ELISA^5^. These findings demonstrate that RmAb158-scFv8D3 enables efficient brain-delivery, which at the time, was ascribed to less bivalent and more monovalent mTfR-binding^5^. However, recent data suggest that at least a portion of RmAb158-scFv8D3 still binds bivalently to mTfR at high concentrations^16^. In the present study, we designed and produced a new set of RmAb158-8D3 antibodies with linkers of different lengths between the scFv8D3 and the antibody’s light chain, with the aim being to improve TfR-mediated transcytosis across the BBB. Our results show that varying the linker length in RmAb158-scFv8D3 variants did not change their apparent affinity to TfR, but improved BBB transcytosis of the shorter-linker variants − 2 aa and 1 aa at higher antibody concentrations in vitro, resulting in transcytosis levels similar to that of the monovalent control. Thus, the shorter-linker variants, despite being bivalent antibodies, appear to crosslink TfR on the cell surface only to a minor degree. A low risk of TfR crosslinking with an antibody that binds both TfR and its therapeutic target bivalently, provides both high BBB transcytosis at low and high antibody concentrations, as well as avidity-enhanced engagement with aggregated targets such as Aβ protofibrils in Alzheimer’s disease.

## Results

### Size, purity and stability characterization of RmAb158-scFv8D3 variants

To study the impact of RmAb158-scFv8D3 linker length on TfR binding and BBB transcytosis, eight RmAb158-scFv8D3 variants were designed that only differed in the length of the flexible amino acid linker between RmAb158 and scFv8D3 (Fig. 1a). A version of 8D3 with the murine IgG2c isotype was considered a bivalent TfR-binding control in all experiments^5^. A construct with one scFv8D3 recombinantly fused to a single chain Fc (scFc)^16^ was used as a monovalent TfR-binding control (Fig. 1b).

The recombinant production of antibodies yielded between 1 and 6 mg protein per liter cell culture. The purity of antibodies was assessed by SDS-PAGE with Coomassie staining (Fig. 1c, Supplementary Fig. S2a,b) and by size exclusion chromatography (SEC) (Supplementary Fig. S1). Under non-reducing conditions, all RmAb158-scFv8D3 variants appeared at their expected size of approximately 200 kDa on SDS-PAGE with a slight upwards shift with increasing linker length. The purity of each antibody was at least 95% as determined by SDS-PAGE and SEC (Supplementary Fig. S1, Fig. S2a). To investigate whether the changes in the linker lengths affected the antibodies’ structural integrity, we employed nano differential scanning fluorimetry (nanoDSF), which determines the antibodies’ change in intrinsic fluorescence at 330 nm and 350 nm over a range of 35 °C to 95 °C (Fig. 1d, Supplementary Fig. S2c, Table S1). Peaks in the first derivate of the fluorescence intensity ratio 350 nm/330 nm are referred to as inflection temperatures and indicate major unfolding events. RmAb158-scFv8D3 with linker lengths between 1 aa and 80 aa, as well as 8D3 were characterized by two inflection temperatures at approximately 67 °C and 79 °C. For the antibody with a -2 aa linker, in which the two N-terminal amino acids of the scFv8D3 had been removed (Fig. 1a), one additional inflection temperature at 60 °C was detected (Fig. 1d). We confirmed by ELISA that the different linker lengths in the RmAb158-scFv8D3 variants did not decrease the antibodies’ affinity to Aβ protofibrils compared with the parental RmAb158 antibody (Supplementary Fig. S3).

## Size, purity and stability characterization of mTfR_Pro119_

**Fig. 2:**
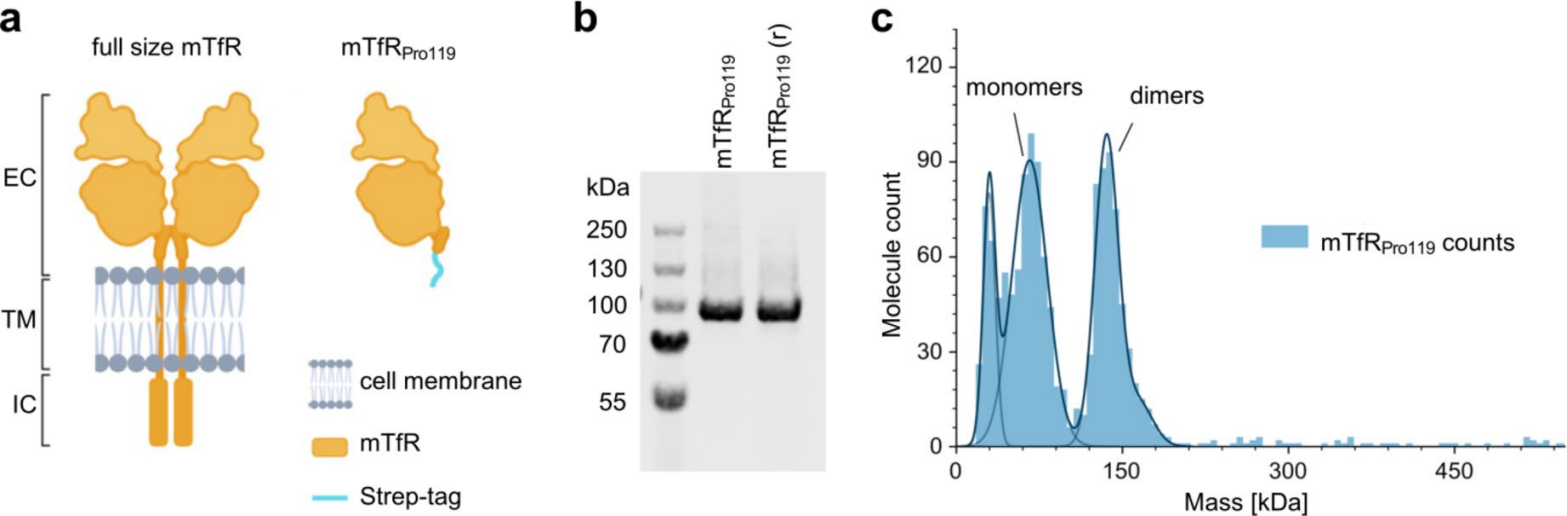
Design and mass distribution analysis of mTfR_Pro119_ used for cell-free in vitro assays. (**a**) Full size murine TfR (mTfR)^7^ consists of an extracellular (EC), transmembrane (TM), and intracellular (IC) domain where the TM domain cannot easily be produced recombinantly in solution. mTfR_Pro119_ comprising of the mTfR ectodomain is commonly used as TfR construct for in vitro assays. (**b**) SDS-PAGE with Coomassie staining of mTfR _Pro119_ under non-reducing and reducing (r) conditions (1 µg protein/lane). The complete gel can be seen in Supplementary Fig. S2d. (**c**) Mass distribution of purified mTfR _Pro119_ at 119 nM in PBS measured by mass photometry. Peak volumes represent counts of molecules of a defined mass (kDa). The first peak likely represents buffer impurities.

For in vitro binding studies of RmAb158-scFv8D3 with TfR, the extracellular domain of murine TfR^7^ (mTfR_Pro119_; Fig. 2a) was produced recombinantly. Its purity was confirmed by SDS-PAGE analysis with Coomassie staining where a strong band just below 100 kDa was in accordance with the theoretical molecular weight of the mTfR_Pro119_ polypeptide (78 kDa) (Fig. 2b). As mTfR is natively dimerizing, mass photometry was carried out to determine the oligomeric state of recombinant mTfR_Pro119_ in solution. Mass photometry results showed that mTfR_Pro119_ in PBS is in a monomer-dimer equilibrium in solution (Fig. 2c). We confirmed by ELISA that holoTf still bound with high affinity to mTfR_Pro119_ at neutral pH (Supplementary Fig. S4).

## Characterization of RmAb158-scFv8D3 variants binding to mTfR_Pro119_ at high coating concentration in ELISA

**Fig. 3:**
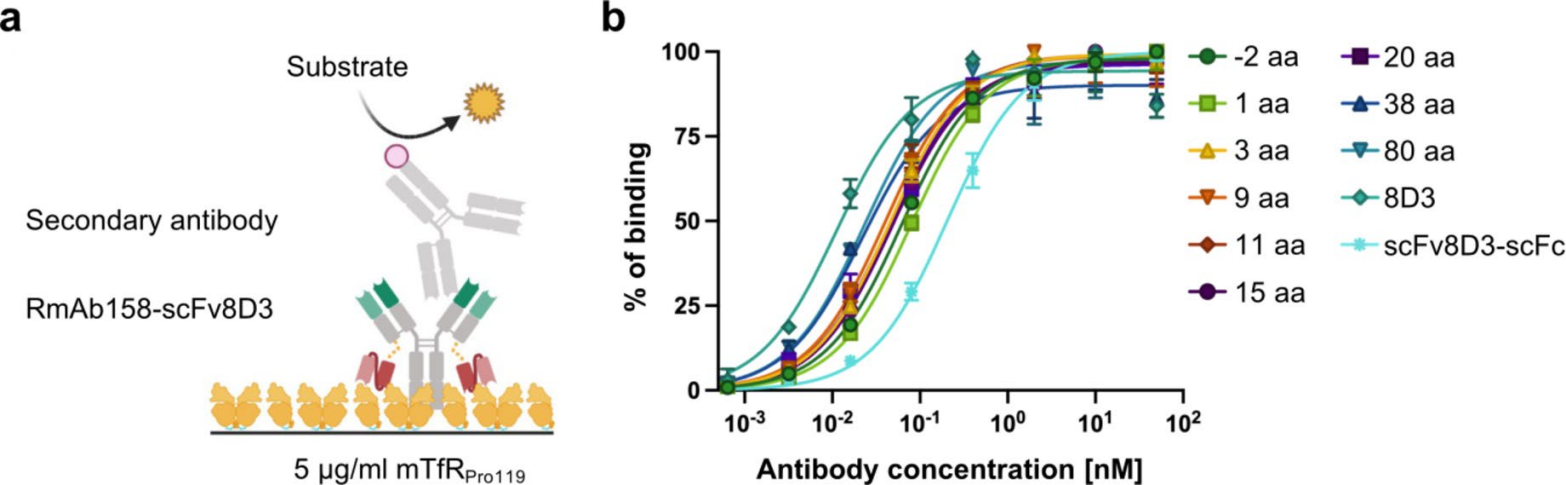
Indirect mTfR_Pro119_ ELISA with RmAb158-scFv8D3 variants and controls binding to high mTfR_Pro119_ coating density. (**a**) Schematic illustration of the indirect ELISA setup with RmAb158-scFv8D3 binding to mTfR_Pro119_. (**b**) ELISA curves of RmAb158-scFv8D3 variants and controls binding to 5 µg/ml mTfR_Pro119_ coating. The data were normalized to maximum binding signal of each antibody. Non-linear regression curves were created using a “one site – specific binding” model in GraphPad Prism. Data points are presented as mean ± SD (*n* = 2). The non-normalized curves can be seen in Supplementary Fig. S5. The raw data can be found in Supplementary Table S3.

To investigate how the linker length affects the binding of the RmAb158-scFv8D2 variants to mTfR_Pro119_, we used an indirect ELISA setup with a high mTfR_Pro119_ coating concentration (Fig. 3a). At this high mTfR_Pro119_ coating density, which was chosen to promote bivalent binding, when possible, we could observe a slight trend of increased binding with increased linker lengths (Fig. 3b, Supplementary Table S2, S3, Figure S5). 8D3 demonstrated the strongest and scFv8D3-scFc the weakest binding to mTfR_Pro119_, which was as expected being that 8D3 and scFv8D3-scFc were considered controls for bivalent and monovalent binding respectively.

## Kinetic evaluation of RmAb158-scFv8D3 variants binding to mTfR_Pro119_ by LigandTracer

**Fig. 4:**
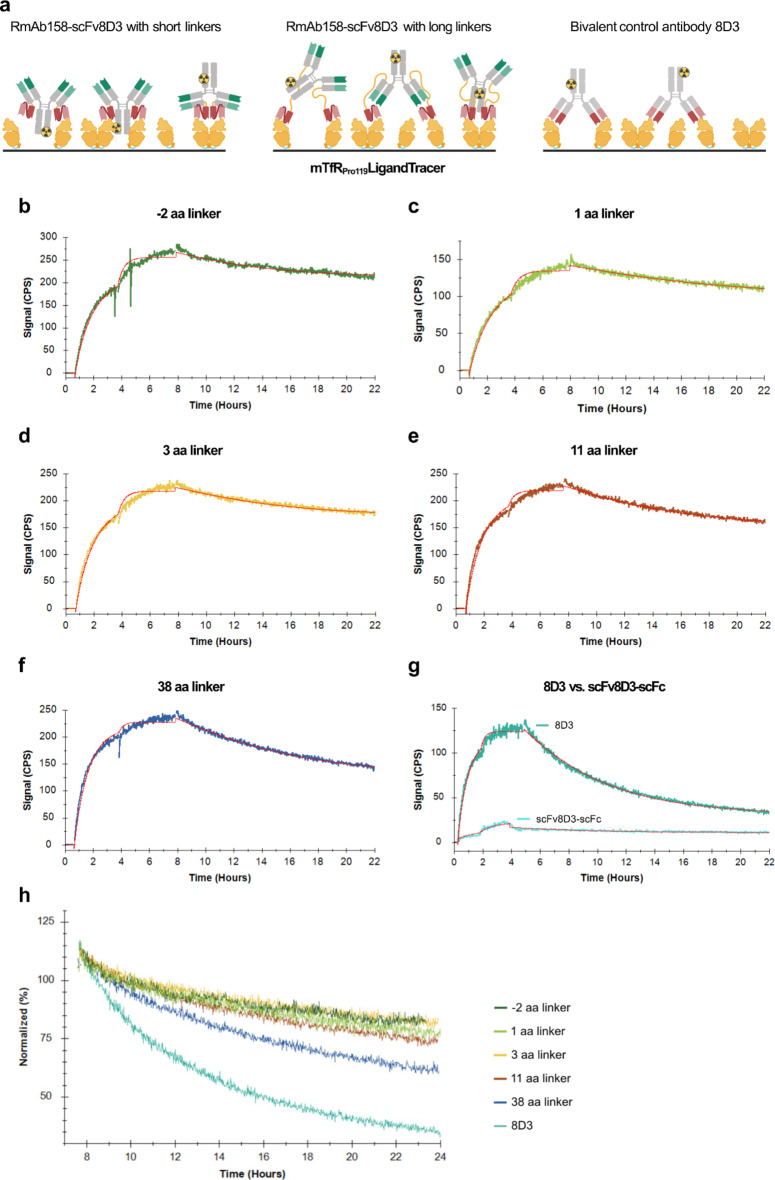
Kinetic evaluation of the interaction of RmAb158-scFv8D3 variants, 8D3 or scFv8D3-scFc with mTfR_Pro119_ by LigandTracer. (**a**) Schematic illustration of the experimental setup with mTfR_Pro119_ coating and ^125^I-labeled RmAb158-scFv8D3 variants or 8D3 added in solution. (**b**–**g**) Interaction curves for ^125^I-labeled RmAb158-scFv8D3 variants, ^125^I-labeled 8D3 or ^125^I-labeled scFv8D3-scFc with mTfR_Pro119_ recorded by LigandTracer. Two consecutive association phases were performed with 10 nM and 30 nM ^125^I-labeled antibody. The subsequent dissociation phase was performed with 30 nM of the respective unlabeled antibody. Kinetic evaluation was done in TraceDrawer using a “one-to-one depletion corrected” fit model. Fitting curves are represented in red. (**h**) Overlay representation of interaction traces with RmAb158-scFv8D3 variants and 8D3 during the dissociation phase. Signal intensities of each curve were normalized to maximum signal at the beginning of the dissociation phase. The raw data can be found in Supplementary Table S4.

To investigate the effect of linker length in the RmAb158-scFv8D3 variants on their binding to mTfR in more detail, we measured the binding kinetics of variants with − 2 aa, 1 aa, 3 aa, 11 aa, and 38 aa linkers by LigandTracer. LigandTracer is particularly suitable for evaluating binding kinetics of very strong interactions with particularly slow dissociation rates, as it allows the observation of the dissociation rate over many hours. We chose this subset with mostly shorter-linker variants as their weaker binding observed by ELISA made them appear more promising for the purpose of improved BBB-transcytosis, whereas the 38 aa linker variant was included as one example of a long linker variant. As before, the antibodies scFv8D3-scFc and 8D3 were used as monovalent and bivalent controls, respectively. A coating of mTfR_Pro119_ was applied as the stationary phase of the assay and the radiolabeled antibodies were added in solution as the mobile phase (Fig. 4a). We confirmed by ELISA that radiolabeling did not affect the antibodies’ affinity to mTfR_Pro119_ (Supplementary Fig. S6). To distinguish monovalent vs. bivalent binding, the dissociation phase was carried out in the presence of an equimolar concentration of unlabeled antibody. Otherwise, the ratio between mono- and bivalently bound antibodies would change when more receptors become available for bivalent binding as dissociation progresses.

A “one-to-one depletion corrected” model was applied, resulting in a good fit to the interaction curve during the first association phase and the dissociation phase for all tested antibodies (Fig. 4b-g, Supplementary Table S4). This model takes into account that the free ligand concentration in solution reduces during the course of the experiment due to target binding which is especially relevant when working with high target concentrations and strong binders^25,26^. The deviation between fitted curves and measured data seen in the second association phase is likely due to the decreasing number of (easily) available targets, which may lower the apparent association rate (k_a_) and was not accurately accounted for by the model. The overlay representation of dissociation curves of RmAb158-scFv8D3 variants and 8D3 (Fig. 4h) indicates the fastest apparent dissociation for 8D3, and decreasing k_d_ with decreasing linker length for the RmAb158-scFv8D3 variants. The calculated dissociation rates (k_d_) of all RmAb158-scFv8D3 variants and 8D3 were of a similar magnitude, whereas a > 10-fold higher k_d_ was calculated for the monovalent scFv8D3-scFc (Table 1). Thus, all RmAb158-scFv8D3 variants are likely binding bivalently under the here tested conditions. For the RmAb158-scFv8D3 variants, a trend of decreasing k_d_ with decreasing linker length was observed (Fig. 4h; Table 1), which might point towards differences in the antibodies’ ability to compete with the unlabeled antibodies for their epitope on TfR.

**Table 1:** Kinetic data from interaction of RmAb158-scFv8D3 variants, 8D3 or scFv8D3-scFc with mTfR_Pro119_, with unlabeled antibody added in dissociation phase, recorded by LigandTracer (Fig. 4b–g).

	B_max_ [Signal (CPS)]	k_a_ [M-1s-1]	k_d_ [s-1]	K_D_ [nM]
-2 aa linker	269	2.14E + 04	7.41E-06	0.35
1 aa linker	227	2.16E + 04	7.23E-06	0.34
3 aa linker	143	1.67E + 04	6.40E-06	0.38
11 aa linker	230	2.37E + 04	9.83E-06	0.43
38 aa linker	238	2.73E + 04	1.33E-05	0.49
8D3	130	3.61E + 04	3.44E-05	0.95
scFv8D3-scFc	32	2.48E + 04	2.76E-04	11.2

Data were calculated using a “one-to-one depletion corrected” model with B_max_ as the maximum binding signal at saturation, k_a_ the association rate constant, k_d_ the dissociation rate constant, and K_D_ the equilibrium dissociation constant.

## In vitro BBB transcytosis assay

**Fig. 5:**
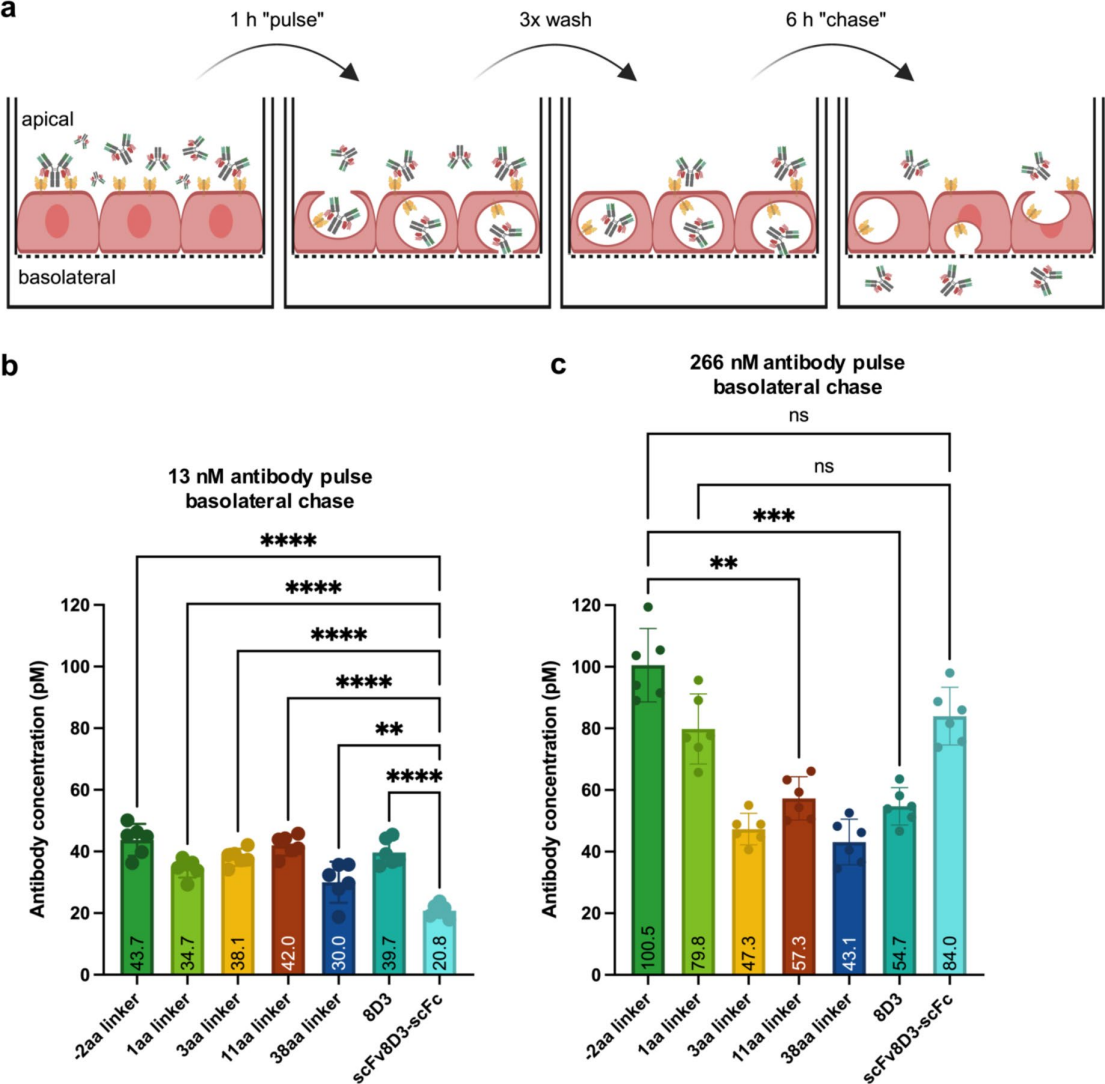
In vitro BBB assay comparing transcytosis efficiency of RmAb158-scFv8D3 variants, 8D3 and scFv8D3-scFc across a BBB endothelial cell monolayer. (**a**) Schematic illustration of experimental setup with cEND cells grown on porous cell culture inserts treated with 13.3 nM or 266 nM antibodies for 1 h, washed and incubated in fresh medium for 6 h. Average antibody concentrations in “chase” samples taken from the basolateral compartment after 6 h were measured by a sandwich ELISA (**b**,**c**). Results are presented as means with 95% confidence intervals. One-way ANOVA with Tukey multiple comparison was applied. (*p < 0.05; **p < 0.01; ***p < 0.001; ****p < 0.0001; ns: *p* > 0.05). (6 wells per condition). The raw data can be found in Supplementary Table S5.

To compare the ability of the RmAb158-scFv8D3 variants to undergo transcytosis through the BBB endothelium, the variants with − 2 aa, 1 aa, 3 aa, 11 aa, and 38 aa linkers were tested in an in vitro BBB model (In-Cell BBB-Trans assay) that was developed as described previously^16,27,28^ (Fig. 5a). We have seen previously that at low concentrations, bivalent constructs pass the BBB more efficiently than at high concentrations, where monovalent constructs cross to a larger extent than bivalent constructs because they do not crosslink TfRs on the endothelial cell surface. For that reason, the in vitro transcytosis assay was run at two antibody concentrations, 13.3 nM and 266 nM, which represent concentrations below and above TfR saturation^27^. At the low concentration of 13.3 nM, all tested RmAb158-scFv8D3 variants showed similar levels of transcytosis as the bivalent antibody 8D3, which were significantly higher than that shown for scFv8D3-scFc (Fig. 5b). At the high concentration of 266 nM, the − 2 aa linker variant, 1 aa linker variant and scFv8D3-scFc achieved the highest level of transcytosis with more than two-fold higher antibody concentrations detected in the basolateral compartment compared to the low treatment concentration (Fig. 5c). The longer-linker variants and the 8D3 antibody only demonstrated a slight increase in the level of transcytosis across the endothelial cells at the high treatment concentration (Fig. 5c).

## Discussion

In this study, we aimed to improve the BBB-passage of the symmetric antibody RmAb158-scFv8D3^5^ by varying the linker length between the therapeutic antibody RmAb158 and its TfR-targeting BBB-shuttle domain scFv8D3. The use of protein drugs for targets located inside the brain is highly restricted by the BBB, but it has been shown that binding to TfR can facilitate the transport of antibodies across the BBB. At elevated administered antibody concentrations, however, bivalent binding to TfR is thought to reduce the transcytosis efficiency through receptor crosslinking and therefore monovalent binding to TfR has been proposed to be preferred^5,6,16,18,20,29^. Hence, our major focus was to investigate to which degree the binding strength of the RmAb158-scFv8D3 variants to TfR was affected by the different linker lengths and which effect this had on the antibodies’ transcytosis across BBB endothelial cells.

One major concern in designing the new RmAb158-scFv8D3 variants was that extremely short linkers could distort the secondary or tertiary structure of both scFv8D3 and the RmAb158 light chain^30^. Long linkers, on the other hand, may be more prone to degradation and aggregation or, if applied in vivo cause immunogenicity^31^. However, we did not detect a significant degree of aggregation in any of the tested antibodies by SEC and the RmAb158-scFv8D3 variants with linker lengths between 1 aa and 80 aa had similar thermal stability. Only the − 2 aa linker variant, where the two C-terminal amino acids of scFv8D3 were removed, demonstrated an additional unfolding event at a lower temperature (59.6 °C), indicating that removing these two amino acids caused a destabilization of the protein under thermal stress. We conclude from these experiments, that varying the linker lengths between 1 aa and 80 aa in RmAb158-scFv8D3 did not affect the antibodies’ thermal stability. We furthermore confirmed by ELISA that the different linker lengths did not negatively affect the affinity of the RmAb158 arms to the therapeutic target Aβ protofibrils (Supplementary Fig. S3). In fact, the shorter-linker variants showed even enhanced binding strength to Aβ protofibrils compared to the parental antibody RmAb158.

To investigate the binding of the new RmAb158-scFv8D3 variants to mTfR_Pro119_, we conducted ELISA and LigandTracer experiments and further evaluated the antibodies’ ability to cross the BBB in the In-Cell BBB-Trans assay.

With the indirect ELISA measuring mTfR binding and the more detailed kinetic analysis by LigandTracer, we observed that all tested RmAb158-scFv8D3 variants had similar apparent affinities to mTfR_Pro119_, in contrast to the lower affinity of the monovalent control, indicating that all RmAb158-scFv8D3 variants can bind bivalently under the given conditions. Nevertheless, several differences seen between the indirect ELISA and LigandTracer results likely arise from differences between the two assay conditions such as end-point vs. real-time measurement, differences in coating concentration and presence of competing unlabeled antibody. In the ELISA, the highest apparent affinity was observed for 8D3, which was as expected because slight conformational changes in the scFv8D3 compared to the original binding sites of 8D3 may affect the epitope recognition negatively. This was supported by the LigandTracer data showing the fastest k_a_ for 8D3. The binding strength of RmAb158-scFv8D3 variants, despite being rather similar, showed a slight decrease with decreasing linker length in the ELISA. Thus, even though all RmAb158-scFv8D3 variants appear to bind bivalently as supported by the LigandTracer results, we speculate that shorter-linker variants may have less options to bind bivalently compared to those antibodies with longer linkers, which can span a greater distance between mTfR_Pro119_ molecules (Fig. 4a). A higher number of longer-linker antibodies binding bivalently might result in a slightly higher apparent affinity of those antibodies compared to the shorter-linker antibodies. This apparent effect of antibodies with longer linkers being able to span between different TfR molecules likely resembles the antibodies’ ability to crosslink TfR molecules on the endothelial cell surface in vivo, suggesting that RmAb158-scFv8D3 with shorter linkers would crosslink TfR to a lesser extent in vivo. One caveat is that we do not know how well the density of mTfR_Pro119_ at a coating concentration of 5 µg/ml on the ELISA plate relates to the TfR density on the endothelial cell surface. Furthermore, it remains unclear whether mTfR_Pro119_ is monomeric or dimeric when applied as coating to the ELISA plate considering that the mass photometry analysis showed that mTfR_Pro119_ is in a monomer-dimer equilibrium in solution (Fig. 2c). The TfR density and presence of monomeric vs. dimeric TfR may have a major impact on the binding behavior of the respective antibodies as these factors might determine the likelihood of an antibody to be able to span between different TfR molecules, i.e. to bind bivalently.

The more detailed kinetic analysis by LigandTracer with a competitive unlabeled binder added in the dissociation phase revealed a trend of decreasing k_d_ with decreasing linker length of RmAb158-scFv8D3 variants. This observation may seem counterintuitive, but could be ascribed to the ability of the unlabeled antibodies to compete for their epitope on the TfR. In that case, an antibody with a long linker has a larger radius of freedom when one arm is not bound than an antibody with a short linker. Thus, more competition with the unlabeled antibodies during unbinding and rebinding antibody arms is likely to happen with a long linker. Due to the very fast k_a_ of all antibodies tested in this study, any dissociation would likely be invisible in the absence of competing, unlabeled antibodies, because a dissociated antibody arm would quickly rebind. However, in the presence of unlabeled antibodies the competition for rebinding leads to a progressive displacement of target-bound, labeled antibodies by unlabeled antibodies and results in a faster drop in signal where more competition is taking place.

We further hypothesize that the shortest linker variants with − 2 aa and 1 aa linkers are able to bind bivalently to the two subunits of one TfR dimer when the two scFv8D3 sit “on top” in between the two antibody arms (Fig. 4a), but are less likely to bind bivalently between two separate TfR subunits unless they are located very close to each other, due to shorter reach. This is in contrast to 8D3 that likely only binds bivalently to separate TfR subunits, but not to two subunits of the same TfR dimer (Fig. 4a). It has been reported that standard IgG antibodies have favored binding to epitopes at a distance of 10 nm, but a strongly reduced probability for binding to epitopes at distances below 8 nm^32^, which is the approximate distance of 8D3 epitopes on TfR (Supplementary Fig. S7). This higher stiffness of the 8D3 antibody at short epitope distances may also explain its rather low B_max_ value, as 8D3 likely “buries” unoccupied epitopes under a bivalently bound antibody and thus reaches target saturation faster (Fig. 4a).

Bivalent TfR binders are thought to cause significant crosslinking of TfR on the cell surface, which reduces the transcytosis efficiency at high, therapeutically relevant antibody doses^16,17,20^. Therefore, monovalent TfR binders are considered to be of advantage for crossing the BBB when administered at high concentrations. At the high antibody concentration applied in our In-Cell BBB-Trans assay, TfR is considered to be saturated with antibodies which reflects the conditions found at a therapeutic antibody dose in in vivo experiments^27^. In contrast, at the low antibody concentration in vitro, TfR is not saturated with antibodies^27^ and bivalent TfR binders have been observed to perform better in crossing the BBB^16^. Despite the relatively similar apparent affinities of all RmAb158-scFv8D3 variants seen by ELISA and LigandTracer, the in vitro BBB assay showed higher transcytosis levels for the − 2 aa and 1 aa linker variants compared to all other bivalent antibodies tested at a high antibody concentration. The transcytosis levels of the − 2 aa and 1 aa linker variants were comparable to that of the monovalent control, which is surprising for antibodies that bind bivalently to TfR. These results suggest that the majority of -2 aa and 1 aa linker antibodies bind bivalently between subunits of one TfR dimer, but much less likely between separate TfR, resulting in a type of bivalent binding that does not affect the transcytosis efficiency negatively. The relative degree of trancytosis comparing different TfR-binders in the In-Cell BBB-Trans assay has previously been shown to translate very well to results obtained from in vivo experiments in mice^16,27^. Hence, based on the improved in vitro transcytosis observed with the short-linker antibody variants here, we expect to also observe an enhanced delivery of these antibody variants to the brain in future in vivo experiments in mice models.

Despite reducing TfR-crosslinking to improve the antibody’s BBB transcytosis efficiency, increasing the antibody’s blood half-life is of strong interest for improved antibody delivery to the brain. Binding of a TfR-targeting antibody to TfR on reticulocytes is considered having a major impact on the antibody’s clearance from the blood^33^. How the altered binding of the short-linker RmAb158-scFv8D3 variant to TfR affects the antibody’s blood half-life in vivo, remains to be investigated, but there is a chance that reduced TfR crosslinking on reticulocytes may also be beneficial for reducing its clearance from the blood circulation.

In conclusion, reducing the linker length in RmAb158-scFv8D3 does not change the apparent affinity of RmAb158-8D3 variants substantially, suggesting that a shorter linker does not force the antibodies to monovalent instead of bivalent binding. However, we hypothesize that the shorter linkers decrease the probability of forming larger crosslinked TfR networks on the endothelial cell surface, as indicated by the increased transcytosis of shorter-linker RmAb158-8D3 variants at high treatment concentrations in the In-Cell BBB-Trans assay. Hence, we suggest that the − 2 aa and 1 aa linker variants bind bivalently mostly to individual TfR dimers or dimers located very close to each other. This way of bivalent TfR binding likely does not affect the transcytosis efficiency negatively, but instead results in equally high transcytosis levels as with a purely monovalent TfR-binder administered at high concentrations. While monovalent TfR-binders are widely thought to be superior in crossing the BBB at therapeutic antibody concentrations, our results provide a new hypothesis about how a bivalent TfR-targeted BBB-shuttle can circumvent TfR-crosslinking and thus result in as good transcytosis levels as that shown for a monovalent BBB-shuttle administered at high concentrations. At low concentrations, on the other hand, the shortest linker RmAb158-scFv8D3 variants appeared to transcytose equally well as other bivalent TfR-binders and thus outperformed the purely monovalent binder at a low antibody dose. Hence, the shortest linker RmAb158-scFv8D3 variants may require lower doses than a purely monovalent TfR-binder to achieve the same brain concentrations, potentially decreasing the risk of side-effects while retaining the same therapeutic effect. Further, the simple, symmetric design of RmAb158-scFv8D3 also allows for easy production and a strong, bivalent engagement with multimeric therapeutic targets, which is highly relevant to achieve antibody-mediated clearance of toxic protein aggregates such as Aβ in Alzheimer’s disease. Thus, compared to Lecanemab, which is the FDA-approved humanized version of RmAb158^34^, our short-linker RmA158-scFv8D3 variants present improved characteristics with the potential to achieve stronger therapeutic effects with lower antibody doses thanks to an active delivery to the brain.

Nevertheless, further in vitro and in vivo studies need to be performed to confirm our hypotheses about antibody-TfR binding stoichiometry and receptor crosslinking and that our conclusions also translate to improved in vivo brain uptake, increased blood half-life and higher therapeutic effects with the short-linker RmAb158-scFv8D3 variants.

## Materials and methods

### RmAb158-scFv8D3 variant design

The RmAb158-scFv8D3 variants were designed by extending or shortening the linker sequence of the previously published RmAb158-scFv8D3^5^, where scFv8D3 are recombinantly fused to the RmAb158 light chain C-terminal (Fig. 1a). To make the − 2 aa linker variant, the two N-terminal amino acids of scFv8D3 were removed and the new scFv8D3 N-terminal was directly fused to the RmAb158 light chain C-terminal (Fig. 1a).

### Protein expression and purification

The genes for all proteins designed in this study were synthesized and cloned into the pcDNA3.4 vector (GeneArt, Regensburg, Germany). Transient transfections into Expi293 cells were carried out as previously described^35^, using polyethyleneimine (PEI) (Polysciences 24765-1) as transfection agent and valproic acid (VPA) (Sigma-Aldrich P4543) as cell growth inhibitor. Vectors for antibodies were added at a ratio of 3:7 of heavy: light chains. Transfected cells were incubated at 37 °C in a shaking incubator with 5% CO_2_ for 7 days. The proteins were subsequently purified from the cell culture medium by affinity purification on a Protein G column (Cytiva GE17-0404-01). Elution fractions containing the protein of interest were concentrated using a 30 K MWCO Amicon Ultra-15 centrifugal filter unit (Millipore UFC9030) and the buffer was changed to PBS using a Zeba spin desalting column 7 K MWCO Zeba spin desalting column (Thermo Scientific 89892).

### SDS-PAGE analysis

Purified proteins were analyzed for purity and size by SDS-PAGE. 1 µg of purified protein was mixed with LDS sample buffer (Invitrogen B0007) with or without 1x Bolt Sample reducing agent (Invitrogen B0009), loaded on a Bolt 4 to 12% Bis-Tris 1 mm protein gel (Invitrogen NW04125) and electrophoretic separation was run at 80 V for 1–2 h. Pre-stained protein marker Plus (Thermo Scientific 26619) or Pre-stained protein marker (Thermo Scientific 26616) was used as a size standard. For total protein detection, PAGE blue protein staining (Thermo Scientific 24620) was applied.

### Structural integrity analysis by NanoDSF

The structural integrity of proteins was analyzed by NanoDSF on a Tycho nt.6 instrument (NanoTemper Technologies, Munich, Germany). The proteins were loaded at equal concentrations into glass capillaries and the fluorescence at 330 and 350 nm was measured while the temperature was increased from 35 °C to 95 °C. Structural changes due to increased temperature cause a shift in the fluorescence intensity as the exposed amount of tyrosine and tryptophane residues changes. The temperature at which a major unfolding event happens is called inflection temperature and is indicated by a peak in the first derivate of the fluorescence intensity ratio 350/330nm.

### Mass photometry

The proportion of mTfR_Pro119_ monomers and dimers in solution was measured by mass photometry on a Refeyn 2MP instrument (Refeyn Ltd., Oxford, UK), calibrated with NativeMark Unstained Protein Standard (Thermo Scientific LC0725). The protein was loaded at 119 nM on the glass surface of the instrument. Each molecule touching the glass surface generates a light scattering signal, which represents one count. The molecular weight of each count was concluded from its proportional relationship to the intensity of the scattering signal.

### Indirect ELISA to measure binding strength of antibodies to TfR

Indirect ELISAs were performed in a high-binding half-area 96-well plate (Corning CLS3690), which was coated with 50 µl of 5 µg/ml mTfR_Pro119_ in PBS overnight at 4 °C. The plate was blocked with 1% BSA (Sigma-Aldrich A7030) in PBS for 2 h shaking at RT. Serial dilutions of RmAb158-scFv8D3 antibody variants and controls were prepared in ELISA incubation buffer (0.1% BSA and 0.05% Tween-20 in PBS), added in duplicates to the plate and incubated for 2 h shaking at RT, followed by goat anti-mouse HRP-conjugated antibody (Sigma-Aldrich 12–349) diluted 1:10,000 in ELISA incubation buffer incubated on the plate for 1 h. A colorimetric signal was generated by the addition of the HRP-substrate K-blue aqueous TMB (Neogen 331177), the reaction was stopped after 1–10 min by adding 1 M sulphuric acid and the absorbance at 450 nm was measured using the TECAN Spark plate reader. Between each step in the protocol, the plate was washed with PBS containing 0.05% Tween-20.

### Labeling of antibodies with Iodine-125

Antibodies were radiolabeled with ^125^I as previously described^36^. In brief, 20 µg of each antibody were incubated with 4 MBq ^125^I (Perkin Elmer Inc., Waltham, MA) and 5 µl of 1 mg/ml Chloramine-T (Sigma Aldrich 857319) for 90 s. The reaction was stopped by adding 10 µl of 1 mg/ml Sodium metabisulphite (Supelco 08982). The buffer of the labeled antibodies was changed to PBS using a 7 K MWCO Zeba spin desalting column (Thermo Scientific 89882) equilibrated with PBS.

### Real-time interaction analysis with LigandTracer

LigandTracer Grey (Ridgeview Instruments, Uppsala, Sweden) was used to record association and dissociation of the antibody variants with mTfR_Pro119_. The instrument is equipped with a low-energy gamma detector above the upper side of an inclined, rotating platform. A Petri dish that is mounted on the platform is coated with the target in a defined “target area” while the opposite site serves as a blank for background measurement. Due to the incline and a rotation of the platform for 180° every 30 s, target area and blank area are alternately incubated in buffer with or without radiolabeled ligand added to the lower part of the dish. The ligand binding is measured by the low-energy gamma detector detecting the radioactivity bound to the respective area.

For the experiments performed in this study, a Petri dish (Sigma-Aldrich P5731) was coated in the target area with 300 µl of 100 nM mTfR_Pro119_ overnight at 4 °C. The next day, the dish was blocked with 1% BSA (Sigma-Aldrich A7030) in PBS for 2 h at RT. The blocking solution was replaced by 2 ml of 0.1% BSA in PBS, the Petri dish was placed in the LigandTracer instrument and the baseline signal was recorded for 10 min. Next, the first association phase was carried out with 10 nM of ^125^I-labeled antibody in 2 ml 0.1% BSA in PBS for 3 h (RmAb158-scFv8D3 variants and scFv8D3-scFc) or 2 h (8D3). For the second association phase, 20 nM ^125^I-labeled antibody was added to the solution to reach a final concentration of 30 nM ^125^I-labeled antibody. After additional 4 h (RmAb158-scFv8D3 variants and scFv8D3-scFc) or 3 h (8D3), the ligand solution was removed, the plate was washed once with 3 ml 0.1% BSA in PBS and the dissociation phase was run with 30 nM unlabeled antibody in 2 ml 0.1% BSA in PBS overnight.

All kinetic evaluations were carried out using TraceDrawer software version 1.9.2 (Ridgeview, Uppsala, Sweden). A “one-to-one depletion corrected” fit model was applied to background-subtracted interaction curves of all antibodies to calculate the k_a_, k_d_ and K_D_. Start values for the parameters in the kinetic evaluation were the default starting guesses for k_a_, k_d_ and B_max_ (starting guess is equal to the highest signal in the measured binding curve). For fitting ligand depletion from solution, the number of targets is estimated and with a 2 ml assay volume that was set constant, the starting guess for the number of TfR in the Petri dish was set to 1.8e^13^.

### In vitro BBB assay

The antibodies’ ability to cross through endothelial BBB cells was evaluated using an In-Cell BBB-Trans assay^27^. Porous cell culture inserts (PCI) (Greiner Bio-One 662640) were coated with 100 µg/ml Collagen Type IV (Corning 354233) and 100 µg/ml fibronectin (Sigma-Aldrich F2006). Murine cerebral endothelial cells (cEND cells, ABM T0290) were seeded in PCI at a density of 9 × 10^4^ cells/well in complete cEND medium (DMEM with 10% FBS, 1x non-essential amino acids, 1x Glutamax and Penicillin/Streptomycin 100 U/ml; all reagents purchased from Gibco). The cells were incubated for at least 4 h at 37°C with 5% CO_2_ to enable attachment to the cell culture insert membrane. The medium in both apical and basolateral chambers was changed to serum-free cEND medium and the cells were left for differentiation for 3 days at 37°C with 5% CO_2_. For the pulse-chase experiment, the medium in the basolateral chamber was replaced with fresh serum-free medium. The medium in the apical chamber was replaced by serum-free medium containing either 13.3 nM or 266 nM of the antibodies. After a 1 h pulse, both apical and basolateral chambers were washed three times with serum-free medium and incubated for additional 6 h (chase) at 37°C with 5% CO_2_. Samples from both apical and basolateral chambers taken after both pulse and chase steps, as well as from the last wash step, were analyzed utilizing a sandwich ELISA. A high-binding 96-well plate was coated with a goat anti-mouse IgG, F(ab’)_2_ fragment-specific antibody (Jackson Immunoresearch 115-005-006) diluted 1:5,000 in PBS overnight at 4°C. The plate was blocked with 1% BSA (Sigma-Aldrich A7030) in PBS for 1 h shaking at RT. Apical wash, basolateral wash and basolateral chase samples were added undiluted; apical pulse, basolateral pulse and apical chase samples were added at suitable dilutions to the plate. For a standard curve, serial dilutions of each antibody starting from 128 pM were prepared and added in additional wells. All samples/standards were incubated for 2 h shaking at RT. Goat anti-mouse IgG HRP-conjugated antibody (Sigma-Aldrich 12–349) was added at a dilution of 1:5,000 for antibody detection and all further steps were performed as described above. The antibody concentration in the basolateral chase sample was calculated by interpolation of the absorbance values at 450 nm with the respective standard curve (sigmoidal, 4PL). GraphPad Prism 9.5.1 was used for the data analysis.

### Statistical analysis

Data were analyzed for statistical significance with a one-way ANOVA with Tukey multiple comparison and results are presented as means with 95% confidence intervals. Significant p-values are defined as *: *p* < 0.05; **: *p* < 0.01; ***: *p* < 0.001; ns: *p* > 0.05. All statistical analysis was carried out in GraphPad Prism 9.5.1.

## Electronic supplementary material

Below is the link to the electronic supplementary material.

Supplementary Material 1

Supplementary Material 2

## Data Availability

Data is provided within the manuscript or supplementary information files.
