# Supplementary material for: A shorter linker in the bispecific antibody RmAb158-scFv8D3 improves TfR-mediated blood-brain barrier transcytosis in vitro
Source: Sci Rep. 2024 Dec 23;14:30613. doi: 10.1038/s41598-024-83627-6 (PMC11666562; doi:10.1038/s41598-024-83627-6)
Supplement: Supplementary file 1 — Supplementary Material 1 [file 41598_2024_83627_MOESM1_ESM.pdf]

Supplementary Data

A shorter linker in the bispecific antibody RmAb158-scFv8D3 improves TfR-mediated Blood-Brain Barrier transcytosis *in vitro*

Inga Petersen[1*], Jamie I. Morrison[1], Alex Petrovic[1], Neira Babic[1], Nicole Metzendorf[1], Ana Godec[1], Andrés de la Rosa[1], Fadi Rofo[1], Sina Bondza[2,3], Jos Buijs[2,3], Farahnaz Ranjbarian[4], Anders Hofer[4], Dag Sehlin[5], Greta Hultqvist[1*]

[1]*Department of Pharmacy, Uppsala University, Uppsala, Sweden.*
[2]*Ridgeview Instruments AB, Uppsala, Sweden.*
[3]*Department of Immunology, Genetics and Pathology, Uppsala University, Uppsala, Sweden.*
[4]*Department of Medical Biochemistry and Biophysics, Umeå University, Umeå, Sweden.*
[5]*Department of Public Health and Caring Sciences, Uppsala University, Uppsala, Sweden.*

*Corresponding authors:
Inga Petersen. Email: inga.petersen@farmaci.uu.se
Greta Hultqvist. Email: greta.hultqvist@farmaci.uu.se

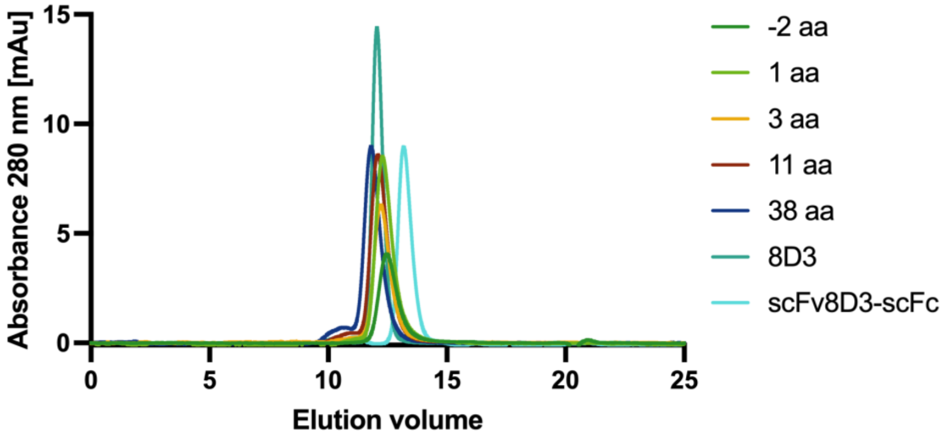

Supplementary Figure S1. Size exclusion chromatography (SEC) with RmAb158-scFv8D3 variants with -2 aa, 1 aa, 3 aa, 11 aa, 38 aa linkers, 8D3 and scFv8D3-scFc. 30 µg of each antibody were injected into a Superdex 200 increase 10/300 GL which was equilibrated with PBS.

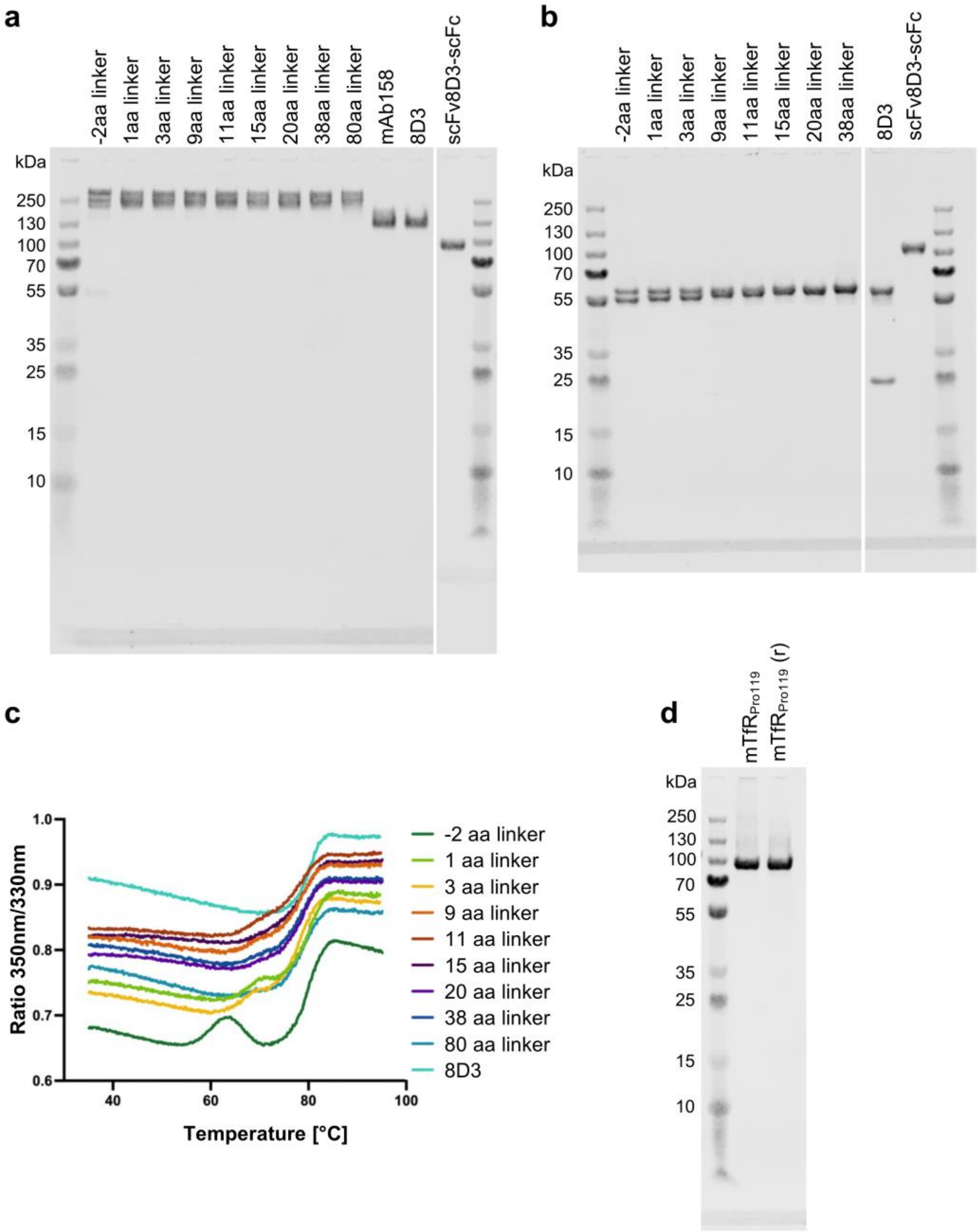

Supplementary Figure S2. (a) Complete image of SDS-PAGE with Coomassie staining of the purified RmAb158-scFv8D3 variants and control antibodies under non-reducing conditions presented in Fig. 1 c. A purity of at least 95% of each antibody was determined by band intensity analysis on the Coomassie-stained SDS-PAGE. (b) Complete image of SDS-PAGE with Coomassie staining of the purified RmAb158-scFv8D3 variants and control antibodies under reducing conditions. (c) Raw data of stability measurement of purified RmAb158-scFv8D3 variants and 8D3 by NanoDSF. Ratio between fluorescence intensities measured at 350 nm and 330 nm. (d) Complete image of Coomassie-stained SDS-PAGE of purified mTfR$_{Pro119}$ presented in Fig. 2 b.

Supplementary Table S1. Inflection temperatures (Ti), representing major protein unfolding events, based on peaks in the first derivate of the ratio between intrinsic fluorescence intensities at 350 nm and 330 nm measured by NanoDSF.

Linkerlength	-2 aa	1 aa	3 aa	9 aa	11 aa	15 aa	20 aa	38 aa	80 aa	8D3
	60.0									
T_i [°C]	67.2	67.7	66.9	68.2	69.0			69.2	67.7	
	80.2	80.4	78.4	78.9	78.7	79.9	79.2	79.2	79.3	80.5

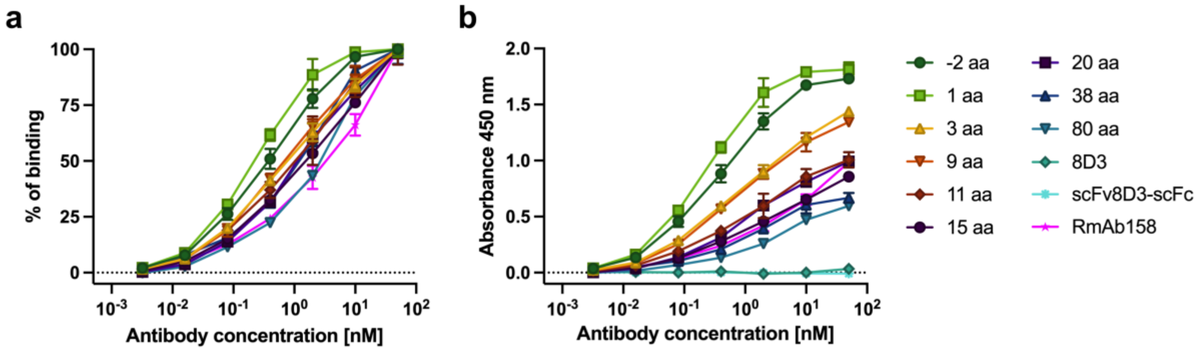

Supplementary Figure S3. Binding curves of an indirect ELISA showing RmAb158-sFv8D3 variants binding to Aβ-42 protofibrils. Curves were normalized to maximum binding signal of each antibody in (a). Non-normalized binding curves are shown in (b). Data points are presented as mean ± SD (n=2). The ELISA plate was coated with Aβ-42 protofibrils and incubated with a dilution series of RmAb158-scFv8D3 variants, scFv8D3-scFc or RmAb158. All RmAb158-scFv8D3 variants bind Aβ-42 protofibrils. The total amount of antibodies binding to Aβ-42 protofibrils appeared to be higher for RmAb158-scFv8D3 variants with shorter linkers compared to those with longer linkers and the parental antibody RmAb158 (b).

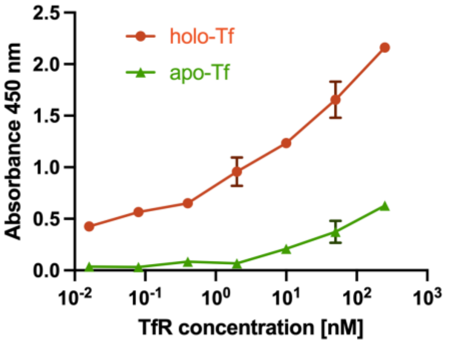

Supplementary Figure S4. Binding curves of an indirect ELISA showing holoTf binding to $mTfR_{Pro119}$. The ELISA plate was coated with holoTf or apoTf and incubated with a dilution series of $mTfR_{Pro119}$ at pH 7.4. The much weaker TfR binding of apoTf compared to the holoTf confirms the specificity of the holoTf binding signal. Data points are presented as mean ± SD (n=2).

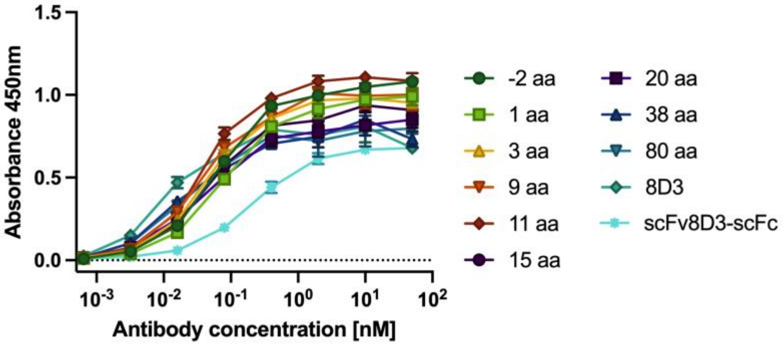

Supplementary Figure S5. Non-normalized binding curves of mTfR$_{Pro119}$ ELISA shown in Fig. 3. ELISA curves of RmAb158-scFv8D3 variants and controls binding to 5 µg/ml mTfR$_{Pro119}$ coating. Data points are presented as mean ± SD (n=2).

Supplementary Table S2. K$_D$ of interaction between RmAb158-scFv8D3 variants and mTfR$_{Pro119}$ determined by indirect ELISA. K$_D$ values were calculated from the normalized binding curves shown in Fig. 3 by non-linear regression curve fit using a "one site – specific binding" model in GraphPad Prism.

Linkerlength	-2 aa	1 aa	3 aa	9 aa	11 aa	15 aa	20 aa	38 aa	80 aa	8D3	scFv8D3
K$_D$ [nM]	0.06	0.08	0.05	0.04	0.04	0.05	0.04	0.02	0.02	0.01	0.2

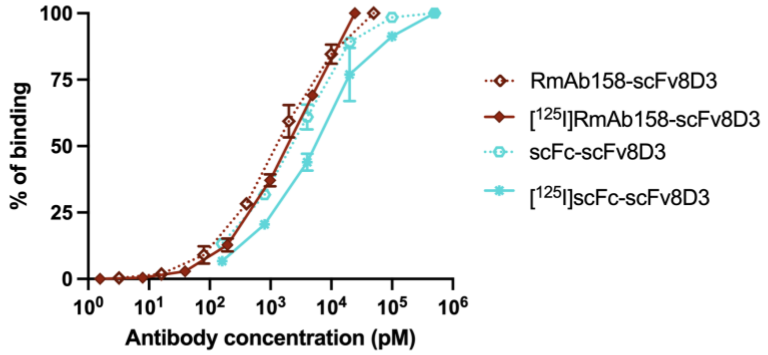

Supplementary Figure S6. Binding curves of an indirect ELISA with ^{125}I-labeled and unlabeled RmAb158 and scFv8D3-scFc binding to mTfR$_{Pro119}$. Only minor differences between the affinity of labeled vs unlabeled antibody to the mTfR$_{Pro119}$ coating confirm that the ^{125}I-labeling did not affect the antibody-TfR$_{Pro119}$ interaction considerably. Data points are presented as mean ± SD (n=2).

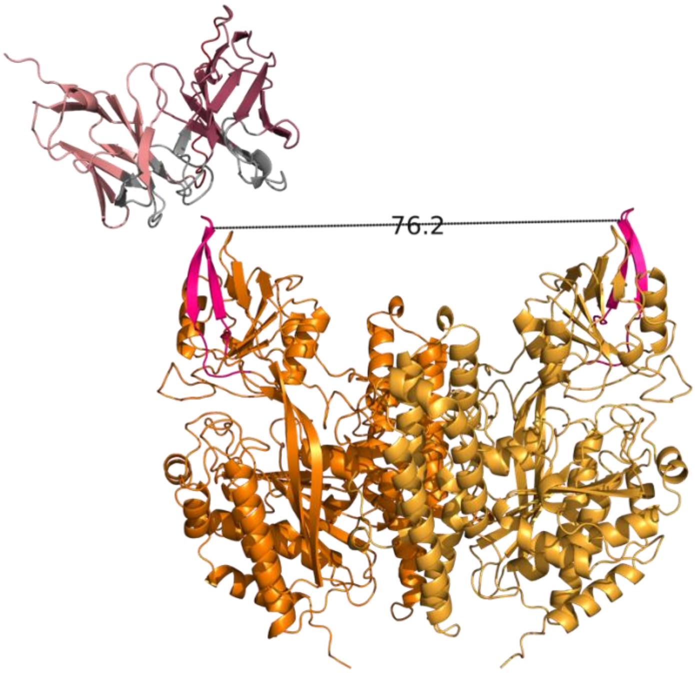

Supplementary Figure S7. Cartoon representation of AlphaFold2-predicted[1,2] structures of the extracellular domain of the mTfR dimer (dimer subunits in dark and light orange) and the scFv8D3 (light chain in light pink, heavy chain in dark purple, CDRs in grey). A distance of 76.2 Å (dashed black line) was measured between the 8D3 epitopes on mTfR (bright pink). The image was generated using PyMOL [3].

Supplementary references

1. Jumper J, Evans R, Pritzel A, Green T, Figurnov M, Ronneberger O, Tunyasuvunakool K, Bates R, Žídek A, Potapenko A, et al. Highly Accurate Protein Structure Prediction with AlphaFold. *Nature*. 2021;596:583–589, doi:10.1038/s41586-021-03819-2.
2. Mirdita M, Schütze K, Moriwaki Y, Heo L, Ovchinnikov S, Steinegger M. ColabFold: Making Protein Folding Accessible to All. *Nat. Methods*. 2022;19:679–682, doi:10.1038/s41592-022-01488-1.
3. Schrödinger, LLC. The PyMOL Molecular Graphics System, Version 3.0.3 2010.
